# Thromboelastography Variables, Immune Markers, and Endothelial Factors Associated With Shock and NPMODS in Children With Severe Sepsis

**DOI:** 10.3389/fped.2019.00422

**Published:** 2019-10-18

**Authors:** Arun Saini, Philip C. Spinella, Steven P. Ignell, John C. Lin

**Affiliations:** ^1^Division of Critical Care Medicine, Department of Pediatrics, Baylor College of Medicine, Texas Children's Hospital, Houston, TX, United States; ^2^Division of Critical Care Medicine, Department of Pediatrics, Washington University School of Medicine, St. Louis, MO, United States

**Keywords:** thromboelastography, coagulopathy, thrombocytopenia, severe sepsis, shock, multiorgan failure, pediatric

## Abstract

**Objective:** Evaluate hemostatic dysfunction in pediatric severe sepsis by thromboelastography (TEG) and determine if TEG parameters are associated with new or progressive multiple organ dysfunction syndrome (NPMODS) or shock, defined as a lactate ≥2mmol/L. We explored the relationship between TEG variables, selective cytokines, and endothelial factors.

**Design:** Prospective observational.

**Setting:** Single-center, quaternary care pediatric intensive care unit.

**Patients:** Children aged 6- months to 14- years with severe sepsis with expected PICU stay for >72 h.

**Interventions:** None.

**Measurements and Main Results:** Twenty-eight children were enrolled with median (IQR) age of 7.3 years (4.4–11.4), PELOD score (study day-1) of 11(1.25–13), and PICU length of stay of 10 days (5–28). TEG-defined hypercoagulable state occurred most commonly in 73% (94/129) of samples, followed by hypocoagulable state in 7.8% (10/129) and mixed coagulation state in 1.5% (2/129) of samples in the study cohort. In contrast, hypocoagulable state occurred most commonly in 66% (98/148) of samples based on standard coagulation parameters. In the seven children who developed shock with NPMODS compared to eight patients with shock without NPMODS and 12 patients with severe sepsis only, we found more profound coagulopathy [thrombocytopenia (*p* = 0.04), elevated INR (*p* = 0.038), low fibrinogen level (*p* = 0.049), and low TEG-G value (*p* = 0.01)] and higher peak of interleukin-6 (*p* = 0.0014) and IL-10 (*p* = 0.007). Peak lactate in the first 5 study days had moderate correlation with standard coagulation assays, TEG parameters, and selective cytokines. Peak lactate did not correlate with markers of endothelial activation. Lowest TEG -G value had moderate correlation with peak IL-10 (ρ −0.442, *p* =0.019), peak VCAM (ρ − 0.495, *p* = 0.007), and peak lactate (ρ −0.542, *p* = 0.004) in the first 5 study days. A combination of TEG-G value and IL-6 concentration best discriminated children with shock and NPMODS [AUC 0.979 (95%CI 0.929–1.00), *p* < 0.001].

**Conclusion:** This exploratory analysis of hemostasis dysfunction on TEG in pediatric severe sepsis suggests that while hypercoagulability is more common, a hypocoagulable state is associated with shock and NPMODS. In addition, TEG abnormalities are also associated with immune and endothelial factors. A larger cohort study is needed to validate these findings.

## Introduction

Pediatric severe sepsis is defined as sepsis with evidence of dysfunction in two or more organ systems with the presence of cardiovascular dysfunction defining septic shock (1). Both severe sepsis and septic shock correlate with activation of immune, endothelial, and hemostatic systems (2–4). Classically, a hypercoagulable state with or without hyperfibrinolysis has been associated with thrombotic microangiopathies caused by diffuse microvascular thrombi or early Disseminated Intravascular Coagulation (DIC) (5). With DIC in particular, as hypercoagulability and clotting factor consumption continues, a hypocoagulable state develops with increased risk of bleeding due to the depletion of coagulation factors and thrombocytopenia (4).

Sepsis-related hypocoagulability has been associated with cardiovascular dysfunction and shock in adults and children (2, 3). Unlike in acute traumatic injury where tissue hypoperfusion and shock have been reported to contribute independently to hypocoagulability and increased risk of death in both adults and children (6–9), the causative mechanisms of how septic shock affects hemostatic dysfunction have not been as well-defined or demonstrated. A more thorough understanding of both the relationship between septic shock and hemostatic dysfunction and also methods to measure this interaction may allow timely intervention and prevention of hemostatic dysfunction with subsequently improved outcomes.

Plasma-based assays including prothrombin (PT), international standardized ratio (INR), and activated partial thromboplastin time (aPTT) have been traditionally used to evaluate hemostatic potential. These tests have several significant limitations: (1) they do not account for the cellular contribution to hemostasis by platelets and red blood cells (RBC); (2) they cannot quantify hypercoagulable states; and (3) they attempt to evaluate in isolation two limited components of the complex enzymatic cascades that result in a continuous balance between clot formation and breakdown. Because of these limitations, investigators and clinicians have begun to study and use viscoelastic assays such as thromboelastography (TEG) to evaluate hypo- and hypercoagulable states in both research and clinical arenas (10). Theoretically, by evaluating the combined contributions of the cellular and plasma protein components on hemostasis, viscoelastic monitoring may provide a more accurate reflection of hemostatic capacity. A recent Cochrane review indicated that the application of a viscoelastic-guided transfusion strategy reduces the amount of bleeding, decreases the incidence of renal injury and may decrease mortality (11). The use of TEG to monitor hemostasis is frequent in some pediatric clinical areas, including cardiovascular surgery, extracorporeal membrane oxygenation (ECMO), and trauma (12–15). However, broader use of TEG remains controversial with unclear indications in patients outside of those with active bleeding or who are receiving systemic anticoagulation.

While there is limited adult and neonatal data on TEG based evaluation of sepsis-induced coagulopathy (SIC) (16–23), to date, there is no published pediatric data. In this study of children with severe sepsis or septic shock, we aimed to describe in detail the incidence of hemostatic dysfunction as defined using TEG variables. We then explored the relationship between both TEG variables and TEG defined hemostatic dysfunction and (1) development of new or progressive multiple organ dysfunction syndrome (NPMODS), (2) development of shock, and (3) changes in markers of endothelial injury and cytokines involved in coagulation activation. Lastly, we explored whether TEG variables, endothelial injury markers, and cytokine levels could be used to create a predictive model for the development of shock and NPMODS.

## Materials and Methods

All patients between ages 6-months and 14- years admitted to a pediatric intensive care unit (PICU) in a large tertiary care children's hospital were screened on a daily basis over an 18 month period from March 2012 through September 2013. Patients >6-months of age were excluded due to the amount of blood that needed to be sampled. Patients above 14 years of age were arbitrarily excluded since they may be more similar to adults regarding their hemostatic system. Patients with severe sepsis or septic shock as defined by Goldstein et al., were eligible for enrollment (1). Additional inclusion criteria were a projected PICU stay of at least another 72 h as predicted by their attending intensivist and absence of restrictions on life-sustaining therapies. Patients with pre-existing hyper- or hypocoagulable disorders, pre-existing liver failure severe enough to cause baseline hypocoagulable states on conventional coagulation assays, or hemoglobinopathies were excluded. Patients receiving systemic anticoagulation or antiplatelet medications were also excluded.

Study enrollment (study day 0) occurred when the patient first met criteria for severe sepsis or septic shock. The following calendar day was assigned as study day 1. Following signed informed consent by the child's guardian, demographic data including age, sex and weight were collected. The severity of illness scores including PIM-2 [Pediatric Index of Mortality-2] and PRISM-3 [Pediatric Risk of Mortality Score-3]) were calculated from the first 24 h of PICU admission. Daily PELOD (Pediatric Logistic Organ Dysfunction) scores were calculated for each day of study participation (study days 0–5). Patients who developed new organ failure per standard definitions (1) during the study period were characterized as having New or Progressive Multiple Organ Dysfunction Syndrome (NPMODS). Shock (inadequate oxygen delivery to meet demand) was defined by a lactic acid concentration of ≥2 mmol/L (24–28). Lactate values were drawn from a free flowing central intravenous catheter or an arterial catheter. We constructed three study groups for comparison in this study; severe sepsis without shock or NPMODS (−S/−NPMODS), severe sepsis with shock but no NPMODS (+S/−NPMODS), severe sepsis with both shock and NPMODS (+S/+NPMODS).

The study period was a total of 7 days. Daily laboratory assessment consisted of complete blood count, chemistry profile, blood gas, lactate, standard coagulation panel (PT, INR, aPTT, fibrinogen, and D-Dimer), and kaolin-activated TEG. If the patient remained in the PICU for longer than 5 days, only interventions and organ dysfunction were noted for an additional two days. Hospital mortality and PICU length of stay (LOS) were recorded.

Coagulation state (hyper or hypocoagulable) was assigned based on all TEG parameters [Reaction (R) time, K time, α angle, maximum amplitude (MA), and G value] (29, 30). We applied our institution's laboratory department defined normal TEG ranges for each parameter. TEG-defined coagulation states were assigned as normal, hypercoagulable, hypocoagulable, or mixed states for each study day. Normal coagulation state was defined as having all TEG parameters for that individual sample falling within normal range. Hypercoagulable state was defined as having one or more TEG parameters from that day's sample demonstrating rapid clot initiation (short R time) or formation (short K time or high α angle) or abnormally high clot strength (high MA or high G). Hypocoagulable state was defined as one or more TEG parameters demonstrating delayed clot initiation (long R time), delayed clot formation (long K time or low α angle), or below normal clot strength (low MA or low G). Mixed state was defined as having an individual TEG panel with some parameters suggesting increased hemostatic function and others consistent with suppressed hemostatic function.

We also measured selected cytokines involved in activation of the coagulation system including interleukin (IL)-1β, IL-6, IL-10, and TNF-α and markers of endothelial injury including intercellular adhesion molecule (ICAM) and vascular cell adhesion molecule (VCAM) by enzyme-linked immunosorbent assay (ELISA) method using commercial available kits on samples drawn during study days 1–5.

Statistical analysis was performed using SPSS v24 (IBM headquarter, New York, NY). Standard descriptive, Chi-square, Fisher's Exact, Wilcoxon Rank Sum, and Kruskal Wallis tests were performed as appropriate. Spearman's correlation test was conducted to determine the correlation between continuous variables. Due to the exploratory nature of this pilot project, we chose not to adjust for multiple comparisons when setting our *p*-value threshold for statistical significance. For plotting the correlation graphs, we removed outliers (>3 standard deviation) on Y-axis. A receiver operating characteristic curve was created to establish predictive models for the development of NPMODS based on hemostatic variables and cytokine levels. The Washington University School of Medicine Institutional Review Board approved this protocol.

## Results

We enrolled 28 patients with a median age of 7.3 years [interquartile range (IQR) of 4.4–11.4] with male predominance 18 (64%) who met criteria for severe sepsis in the PICU. One patient did not have serum lactate levels obtained and was excluded from the groups' comparisons and correlation analysis. Of 27 patients with severe sepsis, 12 (44%) demonstrated neither shock nor NPMODS (−S/−NPMODS), 8 (30%) had shock but not NPMODS (+S/−NPMODS) patients, and 7 (26%) had both shock and NPMODS (+S/+NPMODS). Age, PRISM III scores, PIM-2 scores, and PELOD scores (day 1–5) were similar in the three study groups. Patients with +S/+NPMODS compared to those with -S/-NPMODS and +S/−NPMODS had a longer median PICU length of stay of 48 days (IQR, 24.5–66) vs. 6.5 days (3.2–17.2) and 8 days (4–18), *p* = 0.007, as well as a longer median hospital length of stay of 80 days (46.5–111) vs. 11 days (7–28.5) and 14 days (7–27), *p* = 0.017. Presenting organ dysfunction at the time of meeting severe sepsis criteria were similar in all three groups with respiratory and cardiovascular failure being the most frequently observed. See Table 1. All study subjects survived to hospital discharge.

**Table 1:** Comparison of demographic characteristics among the three study groups.

**Variable**	**−S/−NPMODS^f^ (*n* = 12)**	**+S/−NPMODS (*n* = 8)**	**+S/+NPMODS (*n* = 7)**	***P*-value**
Age in years	8.9 (4.9–13.5)	7.2 (4.2–13)	7.3 (4.1–9.8)	0.27
PRISM^a^-3	7 (4.5–14.2)	7 (4.5–14.5)	11 (11–31)	0.67
PIM^b^-2	−4.2 (-4.8 to−0.8)	−3.9 (-4.6 to−2.0)	−2.8 (-4.6 to−0.7)	0.58
Peak Lactate (mmol/L)	1.2 (0.95–1.55)	4.2 (2.7–5.2)	2.8 (2.2–3.6)	<0.0001
PELOD^c^ score				
Day 1	11 (11–18)	11 (10–12)	13 (10–32)	0.27
Day 2	11 (1–11.25)	11 (1–11)	11 (10–32)	0.41
Day 3	11 (7.75–11)	11 (1.75–11)	13 (10–21)	0.21
Day 4	11 (1–11)	11 (1–11)	10 (2–12)	0.59
Day 5	11 (1–11)	11 (1–11)	11 (10–21)	0.87
Organ failure^d^				0.35
Total	2 (1–3)	2.5 (1–5)	2 (1–6)	
Central Nervous	1	1	1	
Respiratory	9	7	6	
Cardiovascular	11	8	7	
Hepatic	2	1	2	
Renal	0	0	1	
Hematologic	0	4	3	
PICU LOS^e^	6.5 (3.25–17.25)	8 (3.5–20.5)	48 (19–70)	0.007
Hospital LOS	11 (7–28.75)	14 (7–27)	80 (46.5–111)	0.017

*Data are presented as median (interquartile range) except as noted; p-value <0.05 was considered significant*.

a *PRISM – Predictive Risk of Mortality*.

b *PIM – Predictive Index of Mortality*.

c *PELOD – Pediatric Logistic Organ Dysfunction Score*.

d *Organ failure, as defined by Goldstein, et al. (1), at the time of meeting severe sepsis criteria. Data presented as either median (range) or as number of patients with the specific organ system failure*.

e *PICU LOS – Pediatric Intensive Care Unit Length of Stay*.

f *S/NPMODS – Shock/New or Progressive Multi Organ Dysfunction Syndrome*.

On blood samples drawn daily during the five study days, a hypercoagulable state occurred most commonly in 73% (94/129), followed by hypocoagulable state in 7.8% (10/129) samples, and mixed coagulation state in 1.5 (2/129) of samples based on TEG parameters. In contrast, hypocoagulable state based on standard coagulation parameters, occurred in 66% (98/148) of samples. Normal coagulation measurements occurred in 18% (23/129) of samples based on TEG parameters and 34% (50/148) of samples based on standard coagulation parameters.

In the seven patients with +S/+NPMODS, we found increased hypocoagulable state based on lowest platelet count, highest INR, lowest fibrinogen level, lowest TEG- MA and lowest TEG-G value during the first 5 study days compared to those with −S/−NPMODS and +S/−NPMODS as reported in Table 2. There was no difference in the markers of fibrinolysis including D-dimer level and TEG lysis at 30 min between these groups. Also, we found no difference between markers of endothelial injury (ICAM and VCAM) among these groups. On evaluation of specific cytokines, during the first 5 study days we found higher peak serum level of IL-6 and IL-10 in +S/+NPMODS patients compared to −S/−NPMODS and +S/−NPMODS patients.

**Table 2:** Comparison of worst values over five study days of standard coagulation assays, TEG parameters, select cytokines, and endothelial injury markers among study groups.

**Variable**	**−S/−NPMODS^k^*N* =12**	**+S/−NPMODS^k^*N* =8**	**+S/+NPMODS^k^*N* =7**	***P-*value**
Highest WBC^a^ count X10^6^/L	15.4 (10.0–19.6)	20.1 (12.5–27.5)	14.5 (7–29)	0.49
Lowest WBC count X10^6^/L	6.0 (3.95–12.2)	9.0 (7.2–9.7)	3.8 (0.6–8.3)	0.16
Lowest Hb^b^ value in g/dL	8.0 (7.0–9.4)	8.9 (7.0–9.4)	7.4 (6.9–8.5)	0.66
Lowest platelet count X10^9^/L	148 (103–269)	134 (31–160)	34 (22–63)	0.04
Highest INR^c^	1.38 (1.27–1.47)	1.95 (1.42–2.65)	2.18 (1.25–2.39)	0.038
Highest aPTT^d^ in seconds	40.1 (34.8–67.3)	44 (35–63)	45.4 (42.5–55.5)	0.734
Lowest fibrinogen in mg/dL	360 (320–439)	289 (253–356)	289 (232–313)	0.049
Highest D-Dimer in μg/dL	1406 (725–2788)	849 (207–2703)	1897 (1030–9743)	0.16
Longest TEG^e^ R in minutes	5.2 (4.45–7.6)	7.45 (5.2–10)	6.8 (5.6–10)	0.18
Longest TEG K in minutes	1.45 (1.2–1.8)	1.9 (1.2–4.9)	2.1 (2.1–2.6)	0.12
Lowest TEG α in degrees	66 (55–72)	63 (51–68)	60.3 (51–66.4)	0.31
Lowest TEG MA^f^ in minutes	62 (60–70)	60 (41–63)	52.3 (51.1–58.1)	0.01
Lowest TEG G in dynes/cm^2^	8.2 (7.5–11.6)	7.5 (3.6–8.6)	5.5 (5.0–6.9)	0.01
Lowest TEG Lysis in percent	1.5 (0.35–2.35)	0.6 (0–2.7)	0 (0–2.4)	0.37
Highest VCAM^g^ in pg/mL	338416 (288837–437207)	311199 (275462–605595)	469894 (366608–496561)	0.24
Highest ICAM^h^ in pg/mL	424847 (283659–593533)	394866 (269459–685047)	432910 (336291–720209)	0.71
Highest IL^i^−1β in pg/mL	1.54 (0.98–2.5)	1.46 (0.81–182.7)	3.35 (0.55–4.02)	0.78
Highest IL−6 in pg/mL	125 (73–408)	328 (197–498)	5453 (2902–90929)	0.004
Highest IL−10 in pg/mL	14.5 (6.3–32.7)	99 (30–666)	266.8 (49.4–1574)	0.007
Highest TNF^j^ -α in pg/mL	3.6 (2.39–4.8)	4.48 (3.03–1837.2)	4.85 (2.94–12.2)	0.24

*Data are presented as median (interquartile range); p-value <0.05 was considered significant*.

a *WBC – White Blood Cells Count*.

b *Hb –Hemoglobin*.

c *INR – International Normalized Ratio*.

d *aPTT – Activated Thromboplastin Time*.

e *TEG –Thromboelastography*.

f *MA – Maximum Amplitude*.

g *VCAM – Vascular Cell Adhesion Molecule*.

h *ICAM –Intercellular Adhesion Molecule*.

i *IL –Interleukin*.

j *TNF – Tissue Necrosis Factor*.

k *S/NPMODS – Shock/New or Progressive Multi Organ Failure Syndrome*.

**Figure 1:**
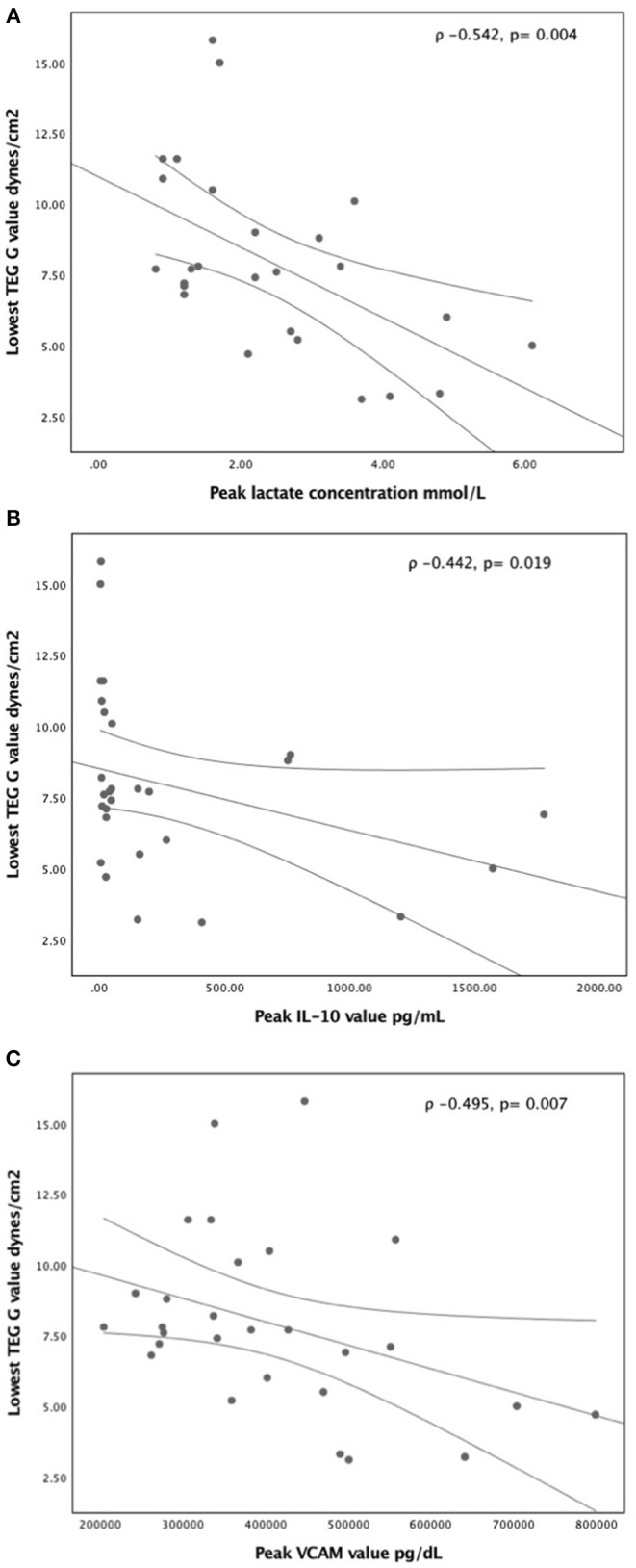
Correlation of lowest TEG- G value with **(A)** peak lactate concentration **(B)** peak IL-10 concentration, and **(C)** peak VCAM concentration in the first five study days. A non-parametric Spearman's correlation coefficient was calculated with *p*-value <0.05 was considered significant.

Peak lactate concentration during the first five study days had significant correlation with the standard coagulation tests of platelet count (ρ −0.543, *p* = 0.003), INR (ρ 0.631, *p* = 0.001), and fibrinogen level (ρ −0.460, *p* = 0.016), but no correlation with aPTT (ρ 0.254, *p* = 0.25) or D-dimer concentration (ρ 0.256, *p* = 0.16) (Supplement Figures 1a–d). For the viscoelastic testing, peak lactate correlated with longest TEG-R (ρ 0.485, *p* = 0.01, longest TEG-K (ρ 0.395, *p* = 0.04), lowest TEG- MA (ρ −0.528, *p* = 0.005), lowest TEG-G (ρ −0.542, *p* = 0.004) (Figure 1A), and highest TEG lysis (ρ −0.460, *p* = 0.01) (Supplement Figures 2a–d). Also, peak lactate concentration correlated with peak IL-6 level (ρ 0.526, *p* = 0.008) and peak IL-10 level (ρ 0.652, *p* < 0.0001), but had no correlation with VCAM (ρ 0.279, *p* = 0.16), ICAM (ρ 0.283, *p* = 0.15), IL-1β (ρ 0.142, *p* = 0.48), and TNF-α (ρ 0.348, *p* = 0.076) (Supplement Figures 3a–d). Of all viscoelastic parameters, only lowest TEG -G value had moderate correlation with peak IL-10 (ρ −0.442, *p* = 0.019) and peak VCAM (ρ −0.495, *p* = 0.007) in the first five study days as illustrated in Figures 1B,C.

**Figure 2:**
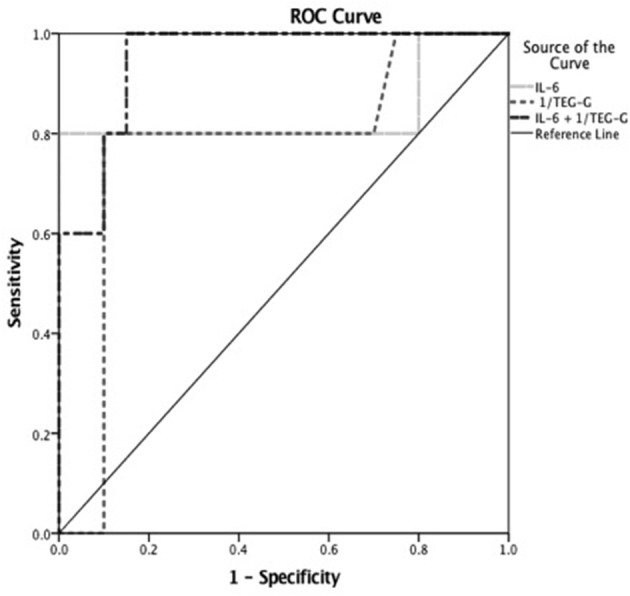
Receiver operating characteristic (ROC) curve to discriminate children with severe sepsis who developed shock with NPMODS (+S/+NPMODS). Worst TEG- G cut off value of <7 dynes/cm^2^ had sensitivity of 81% and specificity of 85.7% with area under the curve (AUC) of 0.776 and 95% CI of 0.574–0.977 (*p* = 0.03). Peak IL-6 cut off value of >1,247 pg/dL had sensitivity of 80% and specificity of 90% with AUC of 0.840 and 95% CI of 0.556–1.000 (*p* = 0.021) to discriminate patients with +S/+NPMODS. A combination of TEG-G value and IL-6 value could best discriminate patients with +S/+NPMODS (AUC 0.979 and 95% CI of 0.929–1.000, *p* < 0.0001) in children with severe sepsis.

On receiver operating characteristic curve analysis to discriminate patients with +S/+NPMODS, a cut off value for TEG- G of <7 dynes/cm^2^ had sensitivity of 81% and specificity of 85.7% with area under the curve (AUC) of 0.776 and 95% confidence interval (CI) of 0.574–0.977 (*p* = 0.03). Similarly, a peak IL-6 cut off value of >1,247 pg/dL had sensitivity of 80% and specificity of 90% with AUC, CI of 0.840, 0.556–1.000 (*p* = 0.021). A peak IL-10 cut off value of >48 pg/dL had sensitivity of 85.5% and specificity of 71.4% with AUC, CI of 0.782, 0.547–1.000 (*p* = 0.028). Among platelet count, INR, TEG-G value, IL-6 and IL-10, a combination of TEG-G value and IL-6 value could best discriminate patients with +S/+NPMODS (AUC 0.979 and 95% CI of 0.929–1.000, *p* < 0.0001) based on non-parametric assumption in our cohort as illustrated in Figure 2.

## Discussion

To our knowledge, this is the first study to report TEG based evaluation of hemostatic dysfunction in children with severe sepsis. Our results indicate that hypercoagulability is the more common coagulation state in children with severe sepsis based on TEG, in contrast to more frequent hypocoagulability state observed on standard coagulation assays. However, children with severe sepsis and +S/+NPMODS are relatively more hypocoagulable based on both TEG and standard coagulation assays compared to children with severe sepsis and −S/−NPMODS or +S/−NPMODS. TEG- G value, a marker of the overall clot formation and stability, decreased with increasing peak lactate concentration, a surrogate marker of oxygen delivery. Among all variables measured, a combination of TEG-G (cut off value of <7 dynes/cm^2^) and IL-6 level (cut off value of >48 pg/dL) could best distinguish children with severe sepsis and +S/+NPMODS in our cohort.

Previous reports have described the varying incidence of hypercoagulable, normal, and hypocoagulable states in adults and neonates with severe sepsis (1–4, 16–23). The incidence of hypercoagulability has been reported in 30–100% of adults with severe sepsis (31). However, although hypercoagulability is frequently described in the early stages of sepsis, development of a hypocoagulable state on TEG is consistently associated with increased morbidity and mortality in adults and neonates with severe sepsis (16–18, 22, 23, 29). Clot strength and formation are largely preserved despite prolongation in clot formation and impairment of fibrinolysis on rotational thromboelastrometry (ROTEM) assays, another viscoelastic assay, in adults with septic shock (23). The kinetics of clot growth utilizing ROTEM assays have been shown to strongly correlate with mortality in adults with severe sepsis (22). Our results suggest that the hypocoagulable state, demonstrated by either delayed clot formation with longer TEG-R and TEG-K times or lower final clot strength with lower TEG-MA and TEG-G values, is more closely associated with higher lactate levels.

Another advantage of TEG is its ability to measure hyper-fibrinolysis. Contrary to other etiologies of DIC in critically ill children, such as trauma, burn, major surgery (7, 12, 32), where hyper-fibrinolysis is frequent (usually defined as >3% lysis at 30 min), patients with severe sepsis frequently show normal or impaired fibrinolysis on TEG (23, 33, 34). As hypo-fibrinolysis is not easy to detect and quantify based on standard viscoelastic and coagulation assays. In a small adult study, a modified urokinase-TEG assay was used to unmask impaired fibrinolysis, the authors reported about half of the patients had impaired fibrinolysis on urokinase-TEG (UK-TEG Lysis at 30 min was lower [70 (29–90) in adults with sepsis vs. 91 (89–93) % in healthy adults, *p* < 0.0001]; also impaired fibrinolysis was associated with increased markers of cellular damage, morbidity, and mortality (33). We also found a negative correlation between the degree of fibrinolysis at 30 min on TEG and peak lactate concentration in our cohort, and none of the patients had hyper-fibrinolysis (>3% lysis at 30 min). These findings support the hypothesis of impaired fibrinolysis in children with severe sepsis, which potentially exacerbate with shock. We need more sensitive and specific assays to quantify fibrinolysis dysfunction in children with sepsis.

Higher pro-inflammatory IL-6 and anti-inflammatory IL-10 levels have been associated with DIC and development of MODS in sepsis (35–37). IL-6 alone or in combination with other inflammatory markers such as procalcitonin, C-reactive protein, and IL-10 has been shown to identify patients with sepsis in both children and neonates (38, 39). IL-6 has been shown to activate various components of hemostasis including endothelial cells, platelets, coagulation proteins and RBCs (35). In an *ex-vivo* model, the addition of IL-6 led to rapid clot formation based on a decrease in TEG-R time but drop in overall clot formation velocity and strength suggestive of alternation in fibrin complexes from fibrinogen and interaction of cellular components such as platelets and RBC in the clot formation (37). In contrast, IL-10 production is increased as a counter to the pro-inflammatory effects of cytokines such as IL-6 and TNF-α. IL-10 has been shown to prevent endotoxin-induced coagulation via inhibition of tissue factor activation (40, 41). In our cohort, higher peak IL-6 and IL-10 levels were associated with the +S/+NPMODS patients, and these patients also developed the hypocoagulable state. The interplay between pro-IL-6 and anti-IL-10 inflammatory cytokines seen in our +S/+NPMODS patients may be a reflection of the ongoing balance between pro- and anti-coagulant stimulants. Thus, our finding of increased hypocoagulable measures in our sickest patients using viscoelastic testing may reflect the counterbalancing effects between IL-10 and IL-6 on overall coagulation status. Viscoelastic tests could give insight into the overall balance between pro-and anti-inflammatory cytokines on sepsis-induced hemostatic dysfunction.

There have also been reports of increased expression of markers of endothelial injury in this patient population. In adults with severe sepsis, ICAM and VCAM levels were found to be elevated in those who developed MODS (42). Similarly, higher levels of ICAM and VCAM were reported in children with severe sepsis and MODS compared to healthy controls and those with severe sepsis only (43). Interestingly in our cohort, we did not find differences between ICAM and VCAM among different groups and only limited associations between VCAM and TEG-G. This may have been related to differences in patient age, disease severity, etiology of infection, and sample size. Thus, further investigation into the interplay between endothelial injury and hemostatic state are needed.

This study had many limitations. First, the patients were enrolled only after fulfilling criteria for severe sepsis, and only if the attending physician predicted a PICU length of stay of at least 72 h. Our patient cohort was thus biased toward those who were perceived to be more severely ill at initial diagnosis of severe sepsis or septic shock. Also, our patient cohort was somewhat older than the typical PICU demographic. This approach also introduced a non-standard length of time between fulfillment of severe sepsis criteria and the time of study day 1 labs that precludes the ability to comment on the timing of onset of hemostatic changes in this study population. Another weakness of this study lies in the non-standardized number of total days of study enrollment. Laboratory studies and data were only collected during the patient's PICU stay, leading to missing data in seven patients, none of whom developed NPMODS. Lastly, because of a small sample size we were unable to account for some important confounding factors including the degree of fluid resuscitation, type and source of infection, age, and use of blood products.

Despite these limitations, in this exploratory study, we were able to demonstrate novel relationships between TEG variables and the development of shock in this population of severe sepsis patients. Furthermore, we found a novel association between TEG parameters and markers of immune function but only limited correlation between TEG parameters and markers of endothelial injury. These findings provide hypotheses generating findings on hemostatic dysfunction based on TEG, correlation of hemostatic dysfunction with lactate, and immune and endothelial alternations in children with severe sepsis.

## Conclusion

This exploratory analysis of hemostasis dysfunction on thromboelastography in children with severe sepsis suggests that while hypercoagulability is more common, a hypocoagulable state is associated with shock and NPMODS. In addition, thromboelastography abnormalities are also associated with alterations in immune and endothelial factors in this population. Larger studies are needed to examine the interactions between oxygen delivery, hemostasis, immune and endothelial function in children with severe sepsis.

## Data Availability Statement

The datasets generated for this study are available on request to the corresponding author.

## Disclosure

PCS research supplies provided by Haemonetics, consultant to Instrumentation Labs.

## Author Contributions

AS performed data analyses, wrote, and reviewed the manuscript. PS conceived and designed the study, provided guidance and supervision with the study conduct, critiqued, and revised the manuscript. SI enrolled the patients, performed the ELISA assays, and reviewed the manuscript. JL conceived and designed the study, enrolled the patients, wrote, and revised the manuscript.

### Conflict of Interest

The authors declare that the research was conducted in the absence of any commercial or financial relationships that could be construed as a potential conflict of interest.

## Supplementary Material

Supplement Figure 1 Correlation of peak lactate concentration with conventional coagulation assays including **(a)** lowest platelet count, **(b)** highest INR value, **(c)** lowest fibrinogen concentration, and **(d)** highest D-dimer concentration. A non-parametric Spearman's correlation coefficient was calculated with *p*-value <0.05 considered significant.

Supplement Figure 2 Correlation of peak lactate concentration with TEG variables including **(a)** lowest TEG-R time, **(b)** longest TEG-K time, **(c)** lowest TEG-MA value, and **(d)** highest TEG-lysis percentage. A non-parametric Spearman's correlation coefficient was calculated with *p*-value <0.05 considered significant.

Supplement Figure 3 Correlation of peak lactate concentration with markers of immune and endothelial activation including **(a)** peak IL-6 concentration, **(b)** peak IL-10 concentration, **(c)** peak VCAM concentration, and **(d)** peak ICAM concentration. A non-parametric Spearman's correlation coefficient was calculated with *p*-value <0.05 considered significant.
