# Supplementary figures and images for: Thromboelastography Variables, Immune Markers, and Endothelial Factors Associated With Shock and NPMODS in Children With Severe Sepsis
Source: Front Pediatr. 2019 Oct 18;7:422. doi: 10.3389/fped.2019.00422 (PMC6814084; doi:10.3389/fped.2019.00422)

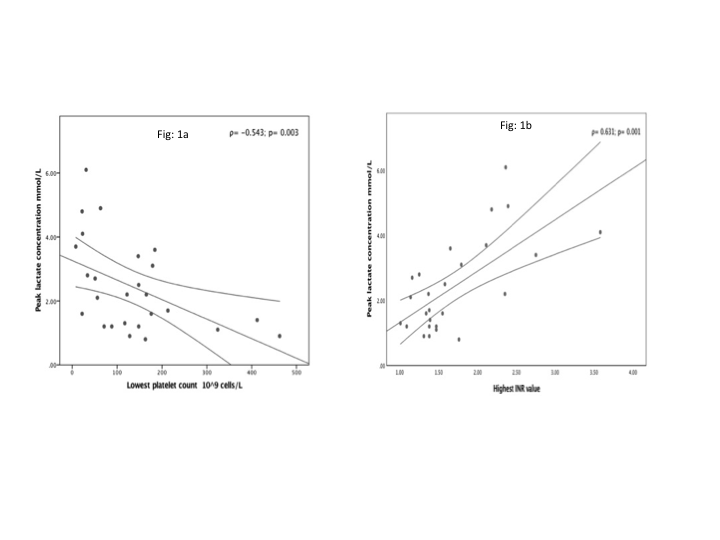

Supplement: Supplementary file 1 [file Image_1.TIFF]

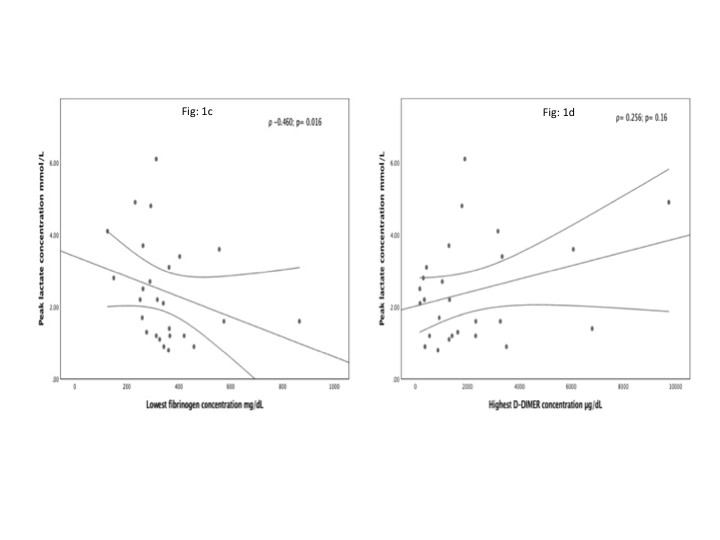

Supplement: Supplement Figure 1 — Correlation of peak lactate concentration with conventional coagulation assays including (a) lowest platelet count, (b) highest INR value, (c) lowest fibrinogen concentration, and (d) highest D-dimer concentration. A non-parametric Spearman's correlation coefficient was calculated with p-value <0.05 considered significant. [file Image_2.TIFF]

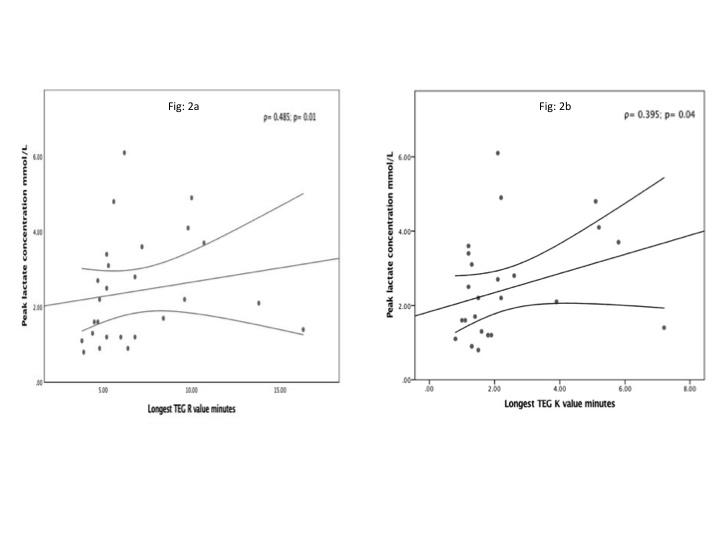

Supplement: Supplementary file 3 [file Image_3.TIFF]

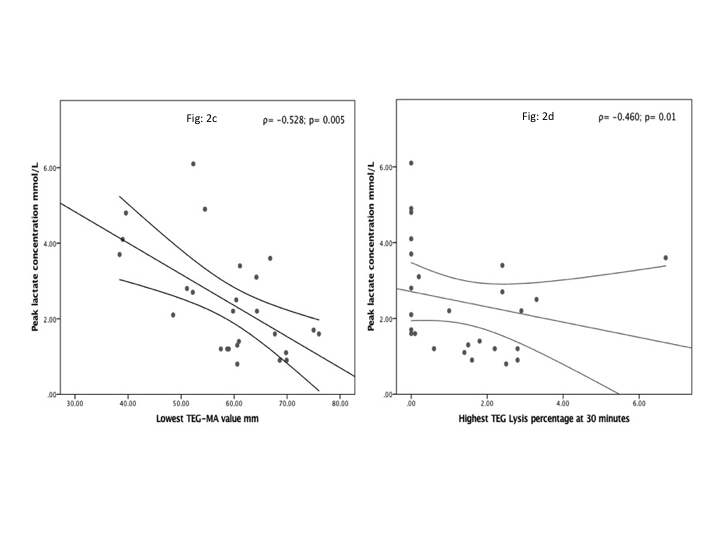

Supplement: Supplement Figure 2 — Correlation of peak lactate concentration with TEG variables including (a) lowest TEG-R time, (b) longest TEG-K time, (c) lowest TEG-MA value, and (d) highest TEG-lysis percentage. A non-parametric Spearman's correlation coefficient was calculated with p-value <0.05 considered significant. [file Image_4.TIFF]

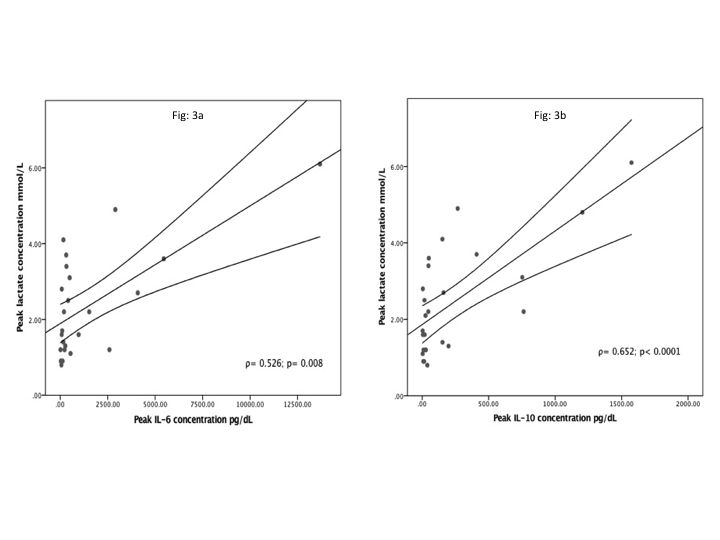

Supplement: Supplementary file 5 [file Image_5.TIFF]

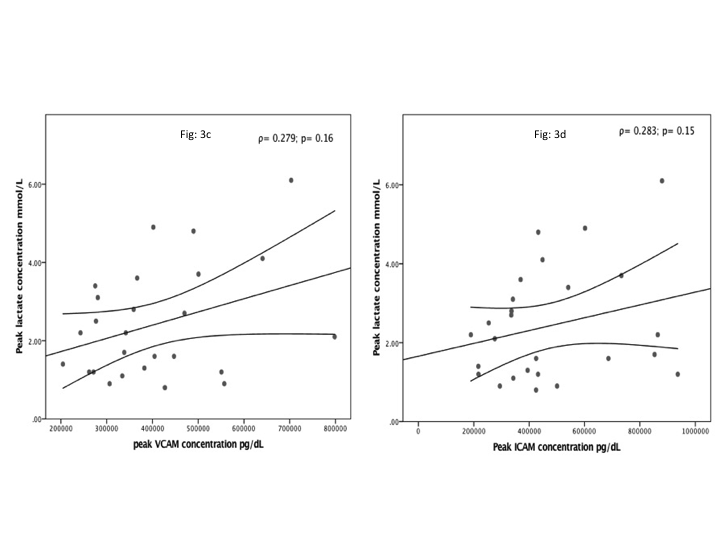

Supplement: Supplement Figure 3 — Correlation of peak lactate concentration with markers of immune and endothelial activation including (a) peak IL-6 concentration, (b) peak IL-10 concentration, (c) peak VCAM concentration, and (d) peak ICAM concentration. A non-parametric Spearman's correlation coefficient was calculated with p-value <0.05 considered significant. [file Image_6.TIFF]
